# Thymoquinone, a potential adjunctive treatment for Ulcerative Collitis

**DOI:** 10.51894/001c.143923

**Published:** 2025-09-30

**Authors:** Syeda Kisa Fatima Zaidi, Adeel Alam

**Affiliations:** 1 Detroit Medical Center Sinai Grace Hospital

## 19

### BACKGROUND

Ulcerative colitis (UC) is a chronic inflammatory bowel disease characterized by persistent inflammation of the colonic mucosa. Current treatments often result in significant side effects and limited long-term efficacy. Thymoquinone (TQ), an active compound derived from Nigella sativa (black seed), has shown promising anti-inflammatory and immunomodulatory properties. This review explores the potential of TQ as an adjunctive treatment for UC, focusing on its effects on molecular pathways implicated in UC pathogenesis.

### METHODS

A comprehensive literature review was conducted using databases such as PubMed. Studies were selected based on their relevance to TQ’s molecular mechanisms, its anti-inflammatory effects, and its application in models of UC and other inflammatory conditions. In vitro and in vivo studies were reviewed to understand the impact of TQ on cytokine production, oxidative stress, and key signaling pathways such as NF-κB and Nrf2.

### RESULTS

TQ exhibits significant anti-inflammatory effects by inhibiting the NF-κB pathway, reducing the production of pro-inflammatory cytokines such as TNF-α, IL-1β, and IL-6. Additionally, TQ activates the Nrf2 pathway, enhancing the expression of antioxidant enzymes and reducing oxidative stress. Animal models of UC have demonstrated that TQ administration leads to decreased histopathological scores, reduced inflammatory cell infiltration, and improved mucosal healing in UC.

### CONCLUSION

Thymoquinone’s ability to modulate the NF-κB and Nrf2 pathways suggests a multifaceted approach to reducing colonic inflammation and promoting mucosal healing. Further clinical trials are warranted to establish its efficacy and safety in human subjects, potentially offering a novel and complementary therapeutic option for UC patients.

